# Neuroprotective effect of diosgenin in a mouse model of diabetic peripheral neuropathy involves the Nrf2/HO-1 pathway

**DOI:** 10.1186/s12906-020-02930-7

**Published:** 2020-04-26

**Authors:** Jinhong Leng, Xiaohua Li, He Tian, Chang Liu, Yining Guo, Su Zhang, Yang Chu, Jian Li, Ying Wang, Ling Zhang

**Affiliations:** 1 Department of Endocrinology, Affiliated Hospital of Liaoning University of Traditional Chinese Medicine, Shenyang, 110032 Liaoning China; 2 Department of Traditional Chinese Medicine Clinical Endocrinology, Liaoning University of Traditional Chinese Medicine Graduate School, Shenyang, 110847 Liaoning China; 3 Department of Histology and Embryology, School of Basic Medicine, Jinzhou Medical University, Jinzhou, 121000 Liaoning China; 4 Department of Endocrinology, The First Affiliated Hospital of Jinzhou Medical University, Jinzhou, 121000 Liaoning China

**Keywords:** Diosgenin, Diabetic peripheral neuropathy, Nrf2, HO-1, Oxidative stress

## Abstract

**Background:**

Diabetic peripheral neuropathy (DPN) is one of the most common chronic complications of diabetes. Diosgenin is a natural steroidal saponin with a variety of beneficial effects, including antidiabetic effects, and is a raw material for the synthesis of carrier hormones. In our study, we aimed to assess the antioxidant effects of diosgenin in diabetic mice.

**Methods:**

Male C57 mice were fed a high-fat diet for 8 weeks and intraperitoneally injected with streptozotocin (STZ) at a dose of 100 mg/kg for 2 consecutive days. Eligible mice were divided into the normal control group (CON), diabetic group (DM), low-dose diosgenin (50 mg/kg) group (DIO50) and high-dose diosgenin (100 mg/kg) group (DIO100). Treatment was started 6 weeks after the induction of diabetes by STZ and continued for 8 weeks. Blood sugar and body weight were monitored dynamically. The behavioural effects of diosgenin were detected by a hot tail immersion test and paw tactile responses. HE staining was used to evaluate edema and degeneration of the sciatic nerve. The levels of SOD, MDA and GPx were tested according to the instructions of the respective kits. The levels of Nrf2, HO-1 and NQO1 were detected by immunofluorescence and Western blotting. Statistical analysis was performed using SPSS, and *P* < 0.05 was considered statistically significant.

**Results:**

Diosgenin decreased the blood glucose levels and increased the body weight of diabetic mice. There was a significant increase in the tail withdrawal latency of diabetic animals, and mechanical hyperalgesia was significantly alleviated after diosgenin treatment. Histopathological micrographs of HE-stained sciatic nerves showed improvement after diosgenin treatment. Diosgenin attenuated the level of MDA but increased the activities of SOD and GPx. Diosgenin increased the expression of Nrf2, HO-1 and NQO1.

**Conclusions:**

Our results demonstrate that diosgenin can ameliorate behavioural and morphological changes in DPN by reducing oxidative stress. The Nrf2/HO-1 signalling pathway was involved in its neuroprotective effects.

## Background

Diabetes is a common and complex endocrine disease that can cause serious complications in multiple tissues, and it has become a serious public health problem worldwide [1, 2]. Diabetic neuropathy is an important factor leading to disability in diabetic patients [3]. It is estimated that in 2015, there were 415 million adults worldwide suffering from diabetes; additionally there are many undiagnosed adults who suffer from impaired glucose tolerance, which is a major risk factor for diabetes [4]. There is a common complication in people with diabetes that is characterized by greater sensitivity to noxious stimuli than that of normal people (hyperalgesia) [5]. Clinically, the symptoms of diabetic peripheral neuropathy seriously affect the quality of life and mental health of patients, so treating these symptoms of DPN in the clinic is a new challenge [6]. Currently, there are very few available therapies for diabetic neuropathy because therapeutic opportunities are limited by many factors, such as serious adverse reactions and ineffectiveness. Therefore, we still need to find a suitable treatment for these complications of neuropathy [7].

Chronic hyperglycaemia develops, oxidative stress is activated, and a series of complex reactions lead to nerve tissue damage, which in turn causes neuropathic pain [8]. There are many opinions on the pathogenesis of diabetic neuropathy. Early reports highlighted the importance of four hyperglycaemic-mediated cellular pathways, including the protein kinase C (PKC), advanced glycation end product (AGE), polyol and hexosamine pathways [9]. Later, it was discovered that these pathways are linked by oxidative-nitrosative stress and that oxidative- nitrosative stress is related in some way to all known aetiologies of diabetic neuropathy [10]. Oxidative stress is one of the main potential mechanisms of painful diabetic peripheral neuropathies. Oxidative stress can lead to neurological damage in a variety of neuropathies, including diabetic neuropathy, Charcot-Marie neuropathy, and acrylamide-induced neuropathy [11–14]. Therefore, we assessed changes in oxidative stress our study of DPN.

Neurons have their own defence system to resist oxidative stress, which includes various enzymatic antioxidant and nonenzymatic antioxidants (superoxide dismutase (SOD), catalase, glutathione S-transferase (GST), glutathione peroxidase (Gpx), glutathione (GSH), various vitamins, etc.) that detoxify reactive oxygen species (ROS) and reduce nerve damage, but this defence system is very weak. In the case of chronic hyperglycaemia, the redox balance in the body is disrupted, resulting in damage to proteins, DNA and cell membranes, which ultimately leads to the impairment of neuronal function [11, 15].

Nrf2 is an important transcription factor that regulates cellular oxidative stress. It is beneficial for ameliorating oxidative stress, promoting cell survival and maintaining redox homeostasis in cells. The Nrf2-ARE signalling pathway initiates the regulation of its detoxification enzymes, such as nucleotide adenosine diphosphate hydrogenase (NADPH), haem oxygenase-1 (HO-1) and quinone oxidoreductase-1 (NQO1), which resist oxidative stress and protect cells [16].

At present, it is necessary to design an effective new compound for relieving pain in diabetic peripheral neuropathy. Chinese herbal plants and their active ingredients are used to manage diabetes mellitus and its complications [17, 18]. Diosgenin is the main component of the Chinese herbal medicine *Dioscorea nipponica Makino*, which is a steroidal saponin in the form of glycoside [19, 20]. It has been found to have many beneficial effects, including hypoglycaemic, [21, 22], cardiovascular protective [21, 23] and hypolipidaemic effects [24]. Diosgenin can also attenuate oxidative damage induced by D-galactose in ageing mice [25]. It also enhances the antioxidant defence system, relieves oxidative stress, and reduces inflammation or apoptosis [23]. Therefore, we hypothesize that diosgenin may prevent or ameliorate diabetic peripheral neuropathy.

Streptozotocin (STZ)-induced diabetic rats develop symptoms of hyperalgesia after being exposed to harmful external stimuli, so they are often used as a model of diabetic peripheral neuropathy and can also be used to test the effect of analgesic drugs [26–28]. In this study, we used male C57 mice with STZ-induced diabetes to evaluate the protective effects of diosgenin on neuropathy in diabetic mice. Behavioural testing and measurements of biochemical markers associated with oxidative stress in sciatic nerves were performed.

## Methods

### Animals and treatment

Eight-week-old male C57 mice were purchased from the Laboratory Animal Center of Jinzhou Medical University. The mice were housed under a 12-h light-dark cycle, food and water were freely available. All procedures were conducted in accordance with the ethical guidelines set up by the International Association for the Study of Pain (IASP) on the use of laboratory animals in experimental research. The animal study was approved by the Animal Care and Use Committee of Jinzhou Medical University.

After being fed a high-fat diet (Casein, Lactic, 30 Mesh:200 g; Cystine, L: 3 g; Sucrose, Fine Granulated: 354 g; Starch, Corn: 315 g; Lodex 10: 35 g; Solka Floc, FCC200: 50 g; Soybean Oil, USP: 25 g; Lard: 20 g; S10026B: 50 g; Choline Bitartrate: 2 g; V10001C: 1 g; Dye, Yellow FD&C #5, Alum. Lake 35–42%: 0.05 g; Total: 1055.05 g) for 8 weeks, the mice were randomly assigned to groups and fasted for 12 h. Then, diabetes was induced by intraperitoneal (i.p.) injection of 100 mg/kg STZ (Sigma, USA) for 2 consecutive days. Immediately prior to injection, STZ was dissolved in sodium citrate buffer (pH 4.5). The control mice were intraperitoneally injected with the same dose of sodium citrate buffer. Blood glucose levels in samples from the tail vein were monitored weekly. Weight was evaluated every week. Animals that exhibited blood glucose levels > 16.7 mmol/L within 1 week after the completion of STZ injection were considered diabetic and were included in the study; these animals showed progression to neuropathy-like symptoms and were tested behaviourally after 6 weeks [29, 30].

Eligible mice were divided into the normal control group (CON), diabetic group (DM), low-dose diosgenin (50 mg/kg) group (DIO50) and high-dose diosgenin (100 mg/kg) group (DIO100) [31]. There were six mice per group, and additional mice were added in the case of death. Diosgenin (Molecular formula: C27H42O3, Molecular weight:414.627. Solarbio Science & Technology Co., Ltd.,) was dissolved in 0.5% CMC-Na solution. The drug was administered intragastrically. The control group was given the same dose of vehicle. Treatment was started 6 weeks after the induction of diabetes by STZ diabetes and continued for 8 weeks. The body weight and blood glucose levels of the mice were monitored continuously. Behaviour and related parameters were tested 24 h after the last intragastric administration.

### Behavioural tests

Before starting the behavioural tests, the mice were habituated to the experimental site for 1 h to prevent the environment from affecting behavioural responses. Behavioural testing was performed 24 h after the last intragastric administration of the drug. To test the behavioural effects of diosgenin in diabetic mice, we used the hot tail immersion test and the Von Frey test.

#### Hot tail immersion test

To detect thermal hyperalgesia in mice, warm water with a temperature of 50 ± 0.5 °C was prepared. The mice were gently restrained with a towel, and the tail was exposed. One third of the tail was quickly immersed in the water, and a stopwatch was used to record the duration of tail immersion and the time at which the tail flick reflex began. The cut-off time was set at 30 s to prevent tissue damage. The experiment was repeated 4 times per mouse, and there was an experimental interval of 5 min. The final results were averaged [32].

#### Paw tactile response test

To detect tactile allodynia in the mice, the Von Frey test was performed. The mice were placed in a cage with a stainless-steel mesh bottom for at least 15 min. A series of Von Frey filaments (range: 0.008-300 g) were then gradually applied vertically to the left hind paw of each mouse so that the filament flexed. Lifting, shaking, or licking of the paws were considered positive reactions. Each mouse was tested 3 times with an interval of at least 10 min. The final results were averaged [32].

### Tissue sample collection

At the end of the experiments, the mice were sacrificed by cervical dislocation under anaesthesia with 3% sodium pentobarbital by intraperitoneal injection. Three mice from each group were fixed with 4% paraformaldehyde via arterial perfusion. The sciatic nerves were isolated quickly and stored in 4% paraformaldehyde solution for tissue slicing. The sciatic nerves of the other three mice in each group were isolated quickly and stored at − 80 °C.

### Haematoxylin-eosin staining

The sciatic nerves stored in 4% paraformaldehyde were dehydrated and embedded in paraffin and then cut at a thickness of 5 μm to prepare slices. The sections were stained with haematoxylin and eosin (H&E), sealed with gum, air-dried and observed under a light microscope (magnification: 40×) to evaluate edema and degeneration of the sciatic nerve.

### Evaluation of SOD, MDA and GPx

The levels of SOD, MDA and GPx in the sciatic nerves of the mice were tested according to the instructions of the respective kits (Jiancheng Bioengineering Institute, China).

### Western blot

The sciatic nerves stored at − 80 °C were placed in prepared RIPA lysis buffer, shredded, sonicated, fully lysed, and centrifuged, and the supernatant was collected for quantitative protein analysis. Then, the samples were diluted to the same protein concentration. Equal amounts of proteins were separated by SDS-PAGE and transferred to a PVDF membrane. After blocking with 3% BSA, the membrane was incubated with primary antibodies against Nrf2, HO-1 and NQO1 (Thermo Scientific, USA) overnight at 4 °C. Then, the cells were washed three times with TBST for 5 min each time. The membrane was incubated for 2 h with the corresponding secondary antibody (Thermo Scientific). The bound antibodies were visualized using a Fusion Chemiluminescence Imager. The relative band densities were quantified by densitometry using ImageJ software.

### Immunofluorescence analysis

The sciatic nerve sections were washed three times with PBS for 5 min at room temperature. They were then permeabilized with 3% Triton-X 100 for 5 min and washed three times with PBS. They were then blocked with goat serum for 2 h and incubated overnight at 4 °C with an Nrf2 primary antibody. The staining methods for HO-1 and NQO1 were the same. The next day, the sections were washed three times with PBS for 5 min each and incubated with a secondary antibody directed against the primary antibody for 2 h, after which they were protected from light. The sections were washed three times with PBS, incubated with DAPI, and covered with coverslips. The slides were observed using a fluorescence microscope.

### Statistical analysis

All the obtained data are expressed as the mean ± SEM, and statistical analysis was performed using SPSS version 22.0. The parameters were analysed by using one-way ANOVA followed by least significant difference (LSD) post hoc test. *P* < 0.05 was considered statistically significant.

## Results

### Diosgenin decreased the blood glucose levels and increased the body weight of diabetic mice

**Fig. 1:**
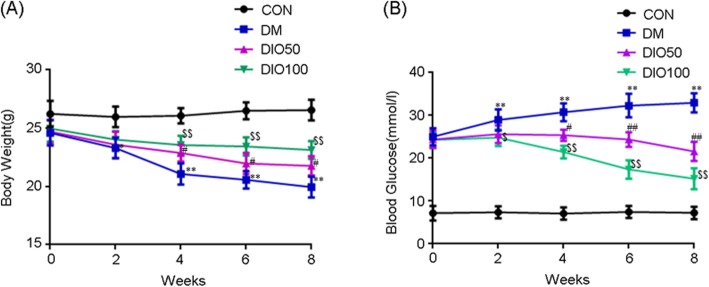
Diosgenin decrease blood glucose and increase body weight in diabetic mice. **a** Body weight of mice in each group. **b** Blood glucose of mice in each group. All data are presented as mean ± S.E.M, *n* = 12. ^*^p < 0.05, ^**^p < 0.01, DM compared with control, ^#^*p* < 0.05, ^##^*p* < 0.01, DIO50 compared with DM, ^$^*p* < 0.05, ^$$^*p* < 0.01, DIO100 compared with DM

During the study period, vehicle-treated diabetic mice showed a significant decrease in body weight at week 8, and compared with the vehicle-treated diabetic mice, the treated mice showed improvements in body weight after the intragastric administration of the two doses of diosgenin for 8 weeks (Fig. 1a). In addition, the diabetic group exhibited a significant increase in blood glucose levels at week 8 compared with week 0, and compared with vehicle-treated diabetic mice, diabetic mice treated with diosgenin presented significantly reduced blood glucose levels at week 8 (Fig. 1b).

### Diosgenin reduced hyperalgesia and allodynia in diabetic mice

**Fig. 2:**
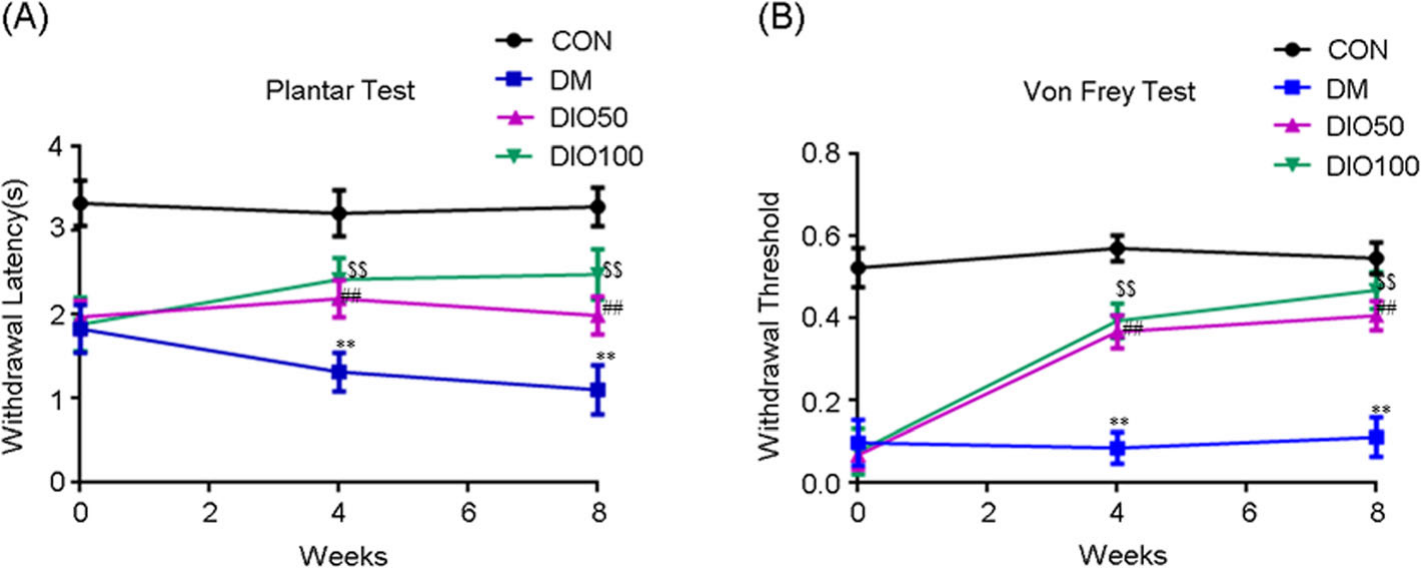
The effect of diosgenin treatment on behavioral performance in diabetic mice. **a** Thermal nociceptive threshold of hot tail immersion test. **b** Mechanical nociceptive threshold of Von Frey test. All data are presented as mean ± S.E.M, *n* = 12.^**^p < 0.01, DM compared with control, ^##^*p* < 0.01, DIO50 compared with DM, ^$$^*p* < 0.01, DIO100 compared with DM

In the hot immersion test, the tail-flick latency of the diabetic animals was significantly lower than that of the normal control animals (*P* < 0.01). There was a significant increase in the tail withdrawal latency of diabetic animals treated with the two doses of diosgenin (*P* < 0.01) (Fig. 2a). Mechanical hyperalgesia was more pronounced in diabetic animals than in normal control animals. The Von Frey test showed that mechanical hyperalgesia was apparent in diabetic animals (*P* < 0.01), and this hyperalgesia was significantly alleviated after diosgenin treatment (*P* < 0.01) (Fig. 2b).

### Diosgenin administration improved histopathological changes in the sciatic nerves of diabetic mice

**Fig. 3:**
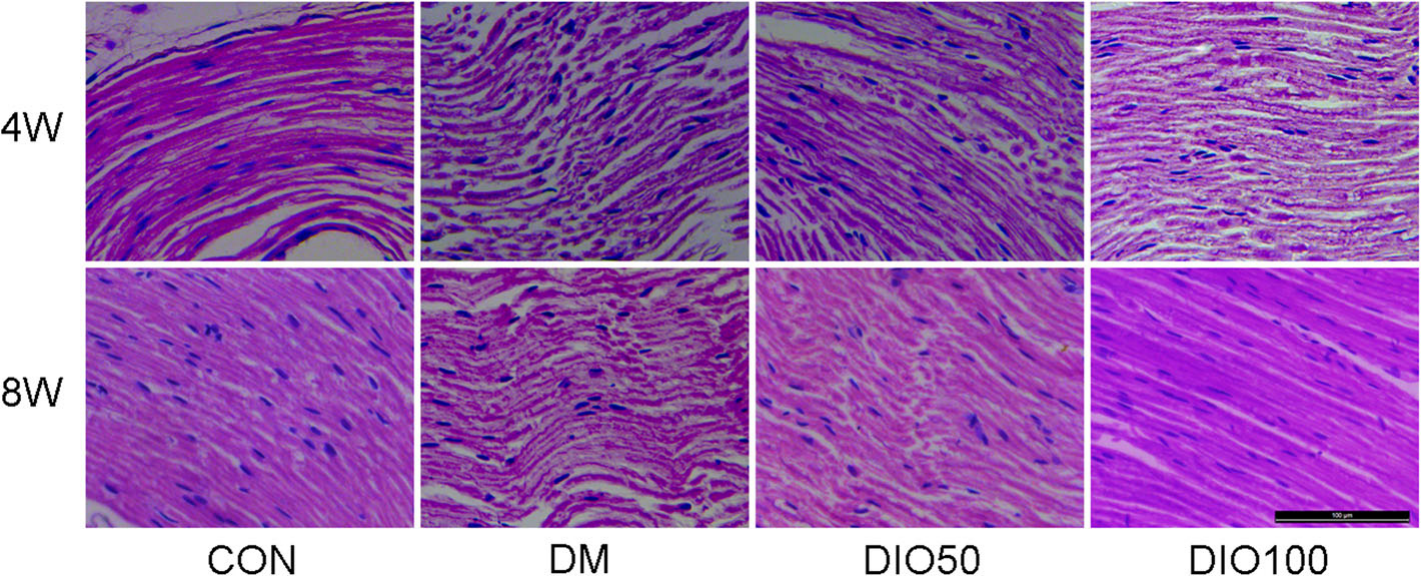
Images of H&E staining of sciatic nerves of mice in each group. In the mice treated with diosgenin 50 mg/kg or 100 mg/kg for 4 W or 8 W, there was improvement with sciatic nerve histopathological changes

Histopathological micrographs showed significant axonal degeneration, myelinolysis, and endometrial edema in the sciatic nerves of DM animals compared to those of the normal control group. There were improvements in axonal degeneration, myelinolysis and endometrial edema in the mice treated with diosgenin (Fig. 3).

### Diosgenin administration reduced oxidative stress in the sciatic nerve of diabetic mice

**Fig. 4:**
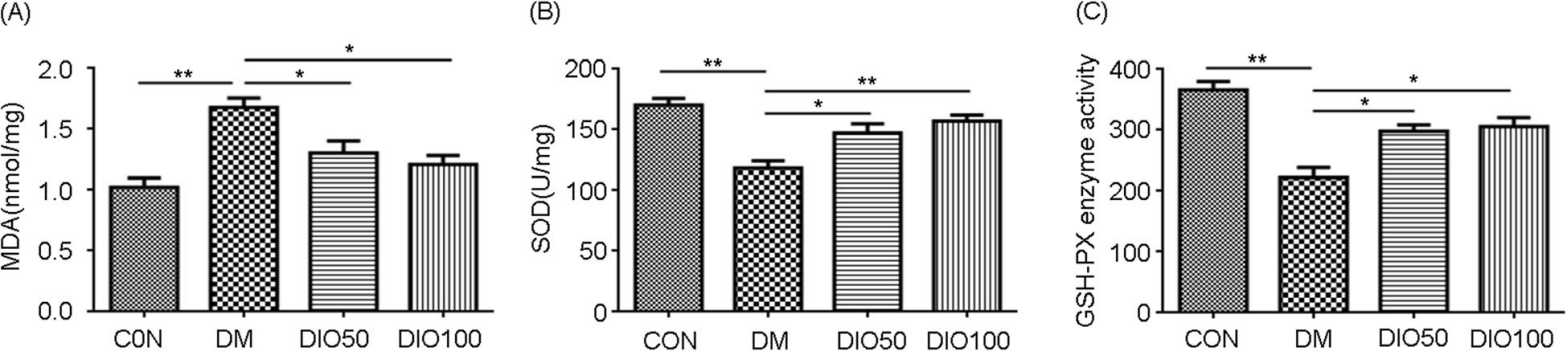
Diosgenin administration reduced oxidative stress in sciatic nerve of diabetic mice. **a** The level of MDA in sciatic nerve. **b** The level of SOD in sciatic nerve. **c** The level of GPx in sciatic nerve. All data are presented as mean ± S. E.M. (*n* = 3), ^*^p < 0.05, ^**^p < 0.01

The level of MDA (a marker of oxidative stress) in the sciatic nerves of diabetic mice was elevated compared with that in the sciatic nerves of normal mice (*P* < 0.01). Diosgenin attenuated the level of MDA, and this effect was dose-dependent (Fig. 4a). The antioxidant enzymes SOD and GPx were inhibited in the sciatic nerves of diabetic mice. The two different doses of diosgenin both increased the activity of antioxidant enzymes (Fig. 4b-c). The results indicated that the administration of diosgenin can restore the beneficial effects of the antioxidant defence system in diabetic mice.

### Diosgenin protected the sciatic nerves of diabetic mice through the Nrf2/HO-1 pathway

**Fig. 5:**
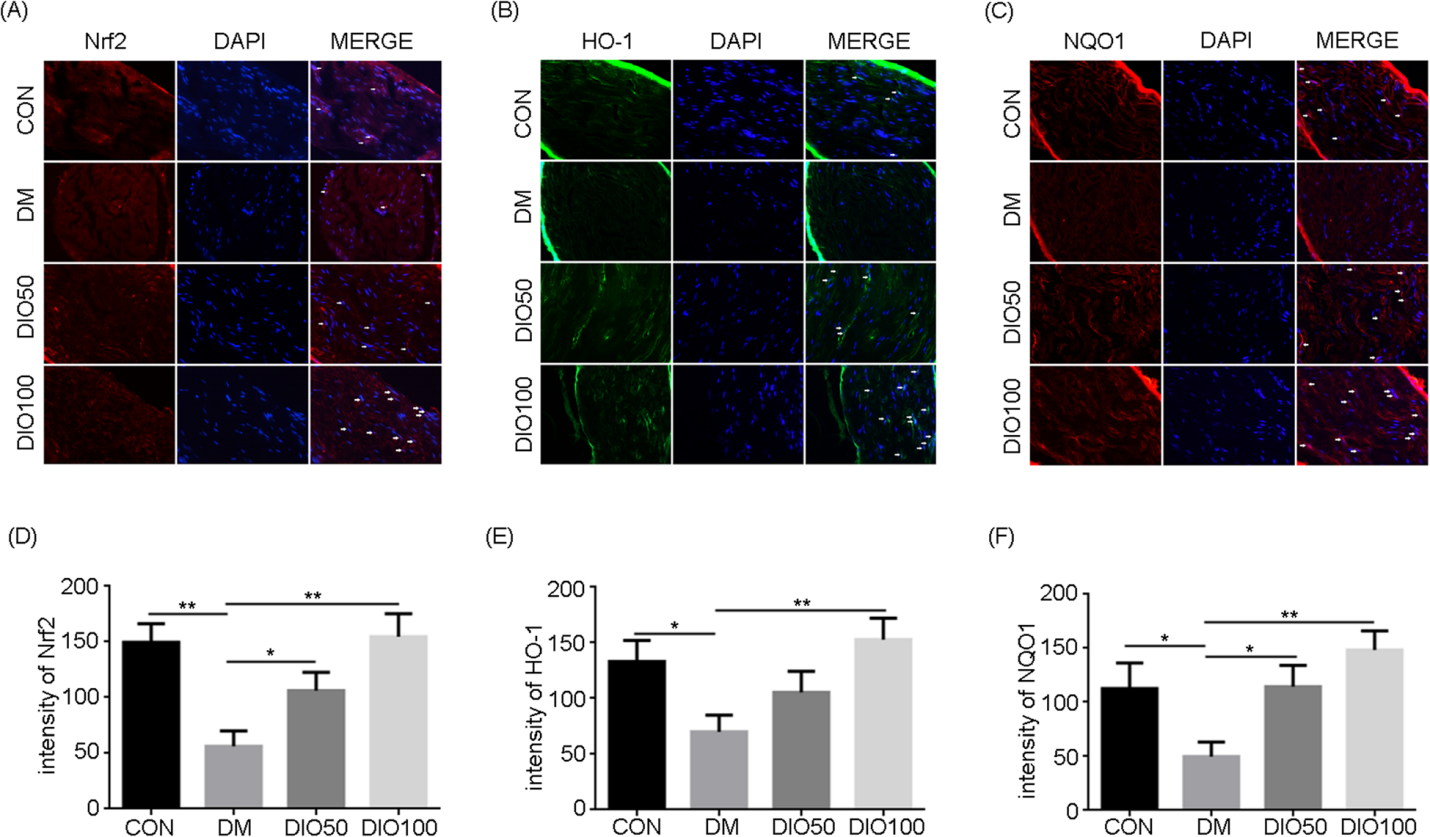
Expression levels of Nrf2, HO-1 and NQO1 in sciatic nerves. Immunofluorescence results of Nrf2 (**a**), HO-1 (**b**) and NQO1 (**c**) in sciatic nerves from different groups. **d** The fluorescence intensity of Nrf2. **e** The fluorescence intensity of HO-1. **f** The fluorescence intensity of NQO1. All data are presented as mean ± S.E.M. (*n* = 3), ^*^p < 0.05, ^**^p < 0.01

**Fig. 6:**
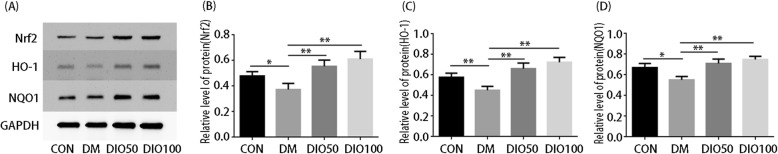
Effect of diosgenin on the Nrf2/ HO-1 signaling pathway. **a** Immunoblot analyses of the protein levels of Nrf2, HO-1 and NQO1 in sciatic nerves treated with diosgenin 50 mg/kg or 100 mg/kg. **b**-**d** Administration of diosgenin increased the protein levels of Nrf2 in diabetic mice, and also increased the levels of HO-1 and NQO1. All data are presented as mean ± S.E.M. (*n* = 3). ^*^p < 0.05, ^**^p < 0.01

To explore the mechanism by which diosgenin resists oxidative stress, the levels of proteins associated with the Nrf2/HO-1 pathway were detected. The results of immunofluorescence staining showed that the expression of Nrf2, HO-1 and NQO1 was decreased significantly in the sciatic nerves of diabetic mice compared to those of normal control mice. However, the fluorescence intensities of Nrf2, HO-1 and NQO1 were increased after the administration of diosgenin (Fig. 5). The Western blot results were consistent with the immunofluorescence staining results. There was a significant decrease in Nrf2 expression in diabetic sciatic nerve compared to normal sciatic nerves (*P* < 0.05). Moreover, decreases in the levels of the downstream cell protective enzymes HO-1 and NQO1 were detected (P < 0.05). The administration of 50 mg/kg and 100 mg/kg diosgenin increased the level of Nrf2 in diabetic mice and increased the levels of HO-1 and NQO1 (*P* < 0.01) (Fig. 6).

## Discussion

DPN is one of the most common complications in diabetic patients and is characterized by disruption of nerve conduction in the peripheral nervous system [33]. The common pathogeneses of DPN are inflammatory damage to myelinated neurons and oxidative stress in endothelial cells [8]. It has been observed in clinical and experimental DPN that the innervation of peripheral nerve tissue is decreased. Because of hypoxia in the endometrium, neuroischaemia, the loss of neurotrophic support, and neurological dysfunction are observed [34]. Studies have shown that a variety of mechanisms, such as adrenergic mechanisms, Na1 currents, opioid neurotransmission and oxidative stress, are involved in the neuropathic pain response in DPN [35–37]. In recent years, many researchers have focused on oxidative stress in peripheral neuropathy. It is an important factor in peripheral neuropathy caused by chemotherapy and diabetic neuropathy [11, 38]. We conclude from this study that the neuroprotective effects of diosgenin are mainly exerted through a reduction in the oxidative stress response and the enhancement of the oxidative defence system. The results confirmed previous findings regarding the therapeutic effects of diosgenin [39, 40].

Histological analysis showed that the biochemical changes in STZ-induced diabetic mice included degeneration of sciatic nerve fibres and endometrial edema. The sciatic nerves of mice treated with diosgenin showed almost normal axons and intact myelin. The beneficial effects of diosgenin observed by histology may be related to its antioxidant capacity. In summary, diosgenin treatment improves tissue changes in the sciatic nerve, possibly due to its antioxidant capacity.

It has been determined that oxidative stress is a major factor in diabetic neuropathy and leads to an abnormal pain response in DPN [8]. The increase of MDA, TBARS, and isoprostanes have been observed in diabetes experimental model [41, 42]. Damage to the antioxidant defence system and hyperoxia cause peripheral nerves to be susceptible to oxidative damage, and neuropathy in diabetic animals can be alleviated by minimizing oxidative damage in the peripheral nerves [43]. ROS can cause damage to peripheral nerves by increasing oxidative stress or attenuating the antioxidant defence systems [11]. They can cause DNA damage and cellular oxidation reactions such as protein oxidation and cell membrane lipid peroxidation [44, 45]. However, the cells themselves have enzymatic and nonenzymatic antioxidant defence systems that detoxify ROS, such as SOD and GPx. However, chronic stress in DPN destroys the antioxidant capacity of cells, leading to the development of abnormal oxidative stress and molecular changes associated with DPN [46]. Previous studies have identified glucose-induced superoxide production as an important part of the pathophysiology of diabetic microvascular complications [10]. High doses of glucose cause NADH flux to increase the intensity of free radical production, which further leads to a series of reactions, such as protein nitration at tyrosine residues and DNA damage [47]. In the present study, the production of MDA by the sciatic nerves was increased in diabetic mice compared with normal control, indicating an increase in oxidative stress. But, diosgenin could ameliorated MDA. Our results also showed that diosgenin significantly increased the levels of SOD and GPx in sciatic nerves. In our study, several changes in the levels of oxidative stress confirmed that diosgenin inhibited oxidative stress in diabetic mice. The reduction in oxidative stress in the sciatic nerve may be attributed to the antinociceptive effect of diosgenin.

Nrf2 is a critical transcription factor of the antioxidant defence system that induces the expression of phase II detoxification enzymes (HO-1, NQO1 and epoxide hydrolase, etc.) [48]. HO-1 has also been found to have potential neurovascular protective properties in diabetic neuropathy [49]. In our study, in the presence of high glucose, DPN mice showed a decrease in the level of Nrf2, which in turn led to decreases in the levels of HO-1 and NQO1. After the administration of diosgenin, the expression of Nrf2 increased in mice, and the trend of HO-1 and NQO1 expression was the same. This shows the beneficial effect of diosgenin on the antioxidant defence system.

## Conclusion

This study focused on improvements in neuropathy elicited by diosgenin in STZ-induced diabetic mice. Our results demonstrate that diosgenin can ameliorate the behavioural and morphological changes observed in DPN by reducing oxidative stress. The Nrf2/HO-1 signalling pathway is involved in the neuroprotective effects of diosgenin.

## Data Availability

The raw data for this study are available upon reasonable request to the corresponding author.
